# Efficacy of Sphincter Control Training and medical device in the treatment of premature ejaculation: A multicenter randomized controlled clinical trial

**DOI:** 10.1371/journal.pone.0257284

**Published:** 2021-09-21

**Authors:** Jesús E. Rodríguez, Jose A. Picazo, Juan C. Marzo, José A. Piqueras, Leandro Reina, Guillermo Hidalgo, Guillermo Tornero

**Affiliations:** 1 Research Area, Murcian Institute of Sexology, Murcia, Spain; 2 Department of Health Psychology, University Miguel Hernández of Elche, Elche, Spain; 3 Urology Service, Rafael Méndez University Hospital, Lorca, Spain; 4 Faculty of Psychology, University of Murcia, Murcia, Spain; National Cancer Institute, UNITED STATES

## Abstract

A new line of treatment for premature ejaculation (PE) based on the use of masturbation aid device in combination with behavioral techniques has emerged in recent years. We report a multicenter randomized clinical trial with a parallel group design to determine the effectiveness of an electronic device called Myhixel I© in the treatment of PE. Forty patients who met the criteria for the diagnosis of lifelong PE, were assigned to two treatment groups completed the Sphincter control training (SCT) program in eight weeks. The only difference between groups was the use of the device. The main measure was the “fold increase” (FI) of the intravaginal ejaculatory latency time (IELT). The geometric means of IELT show, at the end of the treatment at week 8, a superiority of the device group. The mean FI 4.27 (SD 2.59) at the end of treatment for the device group was clearly higher than obtained in the previous clinical trial, in which a specific medical device was not used. No side effects were observed and it required little therapeutic input and no partner involvement. The SCT program in combination with the Myhixel I© is an effective treatment for PE.

## Introduction

Premature ejaculation (PE) is one of the most frequent male sexual dysfunctions [1]. Despite the recent emergence of evidence-based definitions and new PE subtypes, its true prevalence remains ambiguous [1, 2]. This ambiguity surrounding PE is in part due to the difficulty in conducting and interpreting research in the absence of a standardized universal definition that adequately encompasses the characteristics of these patients.

Regarding pharmacological treatment, the evidence so far confirms the efficacy and safety of dapoxetine *(LOE 1a)* and other selective off-label serotonin reuptake inhibitors (SSRIs) *(LOE 1a)*, especially paroxetine and used daily. Some topical anesthetics have also been shown to be effective *(LOE 1b)*, and to date, SSRIs are considered first-line treatments despite their side effects [3, 4].

In relation to behavioral treatments (BTs), the evidence is very limited *(LOE 2b*), although there is an improvement in the results when drugs are combined with BT, in contrast to the exclusive use of drugs [5, 6].

Most studies investigating the efficacy of different behavioral methods do not meet the standards of evidence-based medicine, since they are nonrandomized trials without a control group, use small samples, without adequate follow-up, and/or employ diversity of starting definitions of PE [5, 6].

Despite this limitation, interest in behavioral methods has renewed in the last decade, with the emergence of new underlying hypotheses related with the rol of the bulbospongiosus muscle and the external urethral sphincter in the ejaculatory reflex, and the implementation of new technologies and treatment protocols. Among its advantages are the absence of side effects; the ability to address aspects of PE that medication does not address; the potential to maintain their results as learning, avoiding relapses when stopping the medication; greater access to them; and its implementation without the need to go to the consultation and without the participation of the partner [7, 8].

These new treatment programs combine adaptations of Semans’ (1956) classical behavioral technique of stopping and starting [9] with the use of electronic devices to help masturbation.

In 2019, one randomized controlled clinical trial in which it was implemented a new protocol of cognitive-behavioral treatment for PE called Sphincter Control Training (SCT) were published within this new line of treatment that also provide evidence of its efficacy. The SCT is designed based on hypothesis of Rodriguez (2017) regarding opening of the external sphincter that prevents the formation of the prostate pressure chamber and interferes with the emission phase of ejaculatory reflex [10]. A sex toy not specifically designed for this treatment was used in this first trial [7] to determine the efficacy of SCT.

For this reason and to improve the effectiveness of the method we developed a medical device in collaboration with the company New Wellness concept S.L specifically designed to the SCT.

Consequently, the objective of this new multicenter randomized controlled clinical trial with two parallel groups is to determine whether the latest version of the SCT program by Rodríguez et al., for the treatment of PE, can benefit from the use of a new electronic device especially designed for this program, registered with the FDA as a medical device, which includes new functions such as vibratory stimulation of the penis and the reproduction of intravaginal temperature, given the type of stimulation produced by the masturbation device would make it possible to more easily transfer what was learned to coital relationships by helping men develop greater control, and as a result of using a vibrator during masturbation desensitization can also be achieved in line with this hypothesis, Zamar 2012 [11].

It was hypothesized that the participants of the group that used this new device together with the Sphincter Control Training (SCT) program would achieve a significantly greater improvement in the outcome measures than the participants of the group without a device, as well as better results than in the series of previous studies with this same SCT program in which commercial devices were used [7, 12].

## Materials and methods

A CONSORT-revised 2010 [13] compliant multicenter randomized controlled trial (RCT) parallel group design was used to compare the SCT group with the SCT group plus the device. The attached research protocol was approved by the Research Ethics Committee of the Hospital General Universitario José María Morales Meseguer (José María Morales Meseguer University General Hospital) of Murcia (Spain) (EST: 27/19) and is registered with ClinicalTrials.gov (Identifier: NCT04012437).

The first registration in ClinicalTrials.gov ID: NCT04012437 was on 9 July 2019. The approval of this Ethic committee EST: 27/19 was in 16 June 2019 and we began the patient recruitment in last week July 2019.

The authors confirm that all ongoing and related trials for this intervention are registered.

### Participant recruitment

Prior to the commencement of the trial, power calculations were conducted to determine the minimum sample size informed by the published RCT trial design on PE for psychotherapeutic intervention [14].

We therefore aimed to recruit a minimum sample of 40 participants to allow us to detect population treatment differences with a statistical power of .80 and an alpha level set at P = .05, 1-tail test. The recruitment of patients was developed during July and August 2019 through a health marketing campaign that included radio, press and social networks announcements. We considered all the subjects who complete the study, so the attrition rates were equivalents in both groups and the results don’t return significant modifications.

A total of 50 subjects from all over Spain were recruited, of which 40 completed all phases of the study, with ages between 20 and 52 years of age (M = 34.94 years, SD = 7.826) Table 1.

**Table 1:** Sociodemographic baseline data (mean ± sd) in patients with premature ejaculation who completed all phases of the study.

	GWtD	GWD	*p-* value	*Eta-squared (ηp ^2^)*
**Number of subjects**	21	19		
**Age (years) (mean** ± SD)	32.76 ± 7.10	38.00 ± 7.99	.04	.112
**Duration of the relationship (years)**	8.88 ± 5.76	11.60 ± 8.31	.25	.016
**IELT baseline (SD)**	79.76 ± 33.53	70.81 ± 32.89	.33	.018
**PEDT baseline (SD)**	14.00 ± 2.70	18.07 ± 1.38	.000	.408

GWtD Group: SCT exercise program; Group GWD: SCT exercise program + electronic device SD: Standard deviation. P-value Student’s t-test independent samples

Accepted level of significance p<0.05.

### Inclusion criteria

The study inclusion criteria to be selected were as follows: being over 18 years old, being in a heterosexual relationship for at least the last 6 months, having a score ≥ 11 in the PEDT (Premature Ejaculation Diagnostic Tool) [15] and a mean stopwatch record time self-reported IELT ≤ 2 minutes.

### Exclusion criteria

The study exclusion criteria included history of alcohol abuse or dependence, having received medication or psychological treatment for PE in the last 6 months, suffering from diabetes or habitual use of recreational drugs (except tobacco and caffeine).

### Procedure

All subjects interested in participating were contacted by email or telephone, and questionnaires such as the PEDT, initial interview and sociodemographic data, along with the template for time records, were sent by email. They were also asked for a first stopwatch time record of ejaculatory latency times (IELT) for 2 weeks, and once these data were received, those who met the selection criteria were randomly assigned to the two groups, using the online free version software GraphPad Prism. It randomly scrambles a set number of participants among a set number of treatment slots. So each treatment always gets assigned the same number of participants. Then, they were asked to sign an informed consent before starting the intervention phase.

The subjects were free to leave the study at any time and did not receive financial compensation for participating, although subjects from the device group (GWD) were allowed to keep the device once the study was completed. At the end of the study, subjects without the device (GWtD) were also given the opportunity to use the device to perform the activities. In GWD all the patients excluded after randomization did not send us an informed consent before starting the intervention phase then, no treatment was applied at all and there are no data available after randomization.

In GWD of the six patients excluded after randomization four of them did not send us an informed consent before starting the intervention phase and the other two patients dropped out of the study before they received any treatment and there are no data available after randomization.

Both treatment groups completed the latest version of the 8-week SCT program Table 2.

**Table 2:** Sphincter Control Training program (SCT).

SCT method		
Timeline	Activity	Procedure
Week 1	“Discovering the pelvic floor”	Educational session: Video role of pelvic musculature in the ejaculatory reflex.
		Masturbation 3 times a week paying attention to the role of the pelvic musculature, external urethral and anal sphincter
Week 2,3,4	“Feedback of the external sphincter with stops and starts”	Masturbation 3 times per week with 4 active stops per exercise of maximum 30 seconds relaxing external anal and urethral sphincters at each stop.
Week 5,6	“Feedback of the external sphincter without cessation of stimulation”	Masturbation 3 times per week without cessation of stimulation with 4 moments of relaxation by exercise of the external anal and urethral sphincters before ejaculatory eminence.
Week 7,8	“Feedback of the external sphincter with intercourse movements”	Masturbation 3 times per week without ceasing stimulation with 4 moments of relaxation by exercise of the external anal and urethral sphincters before ejaculatory eminence with intercourse movements.

**Fig 1:**
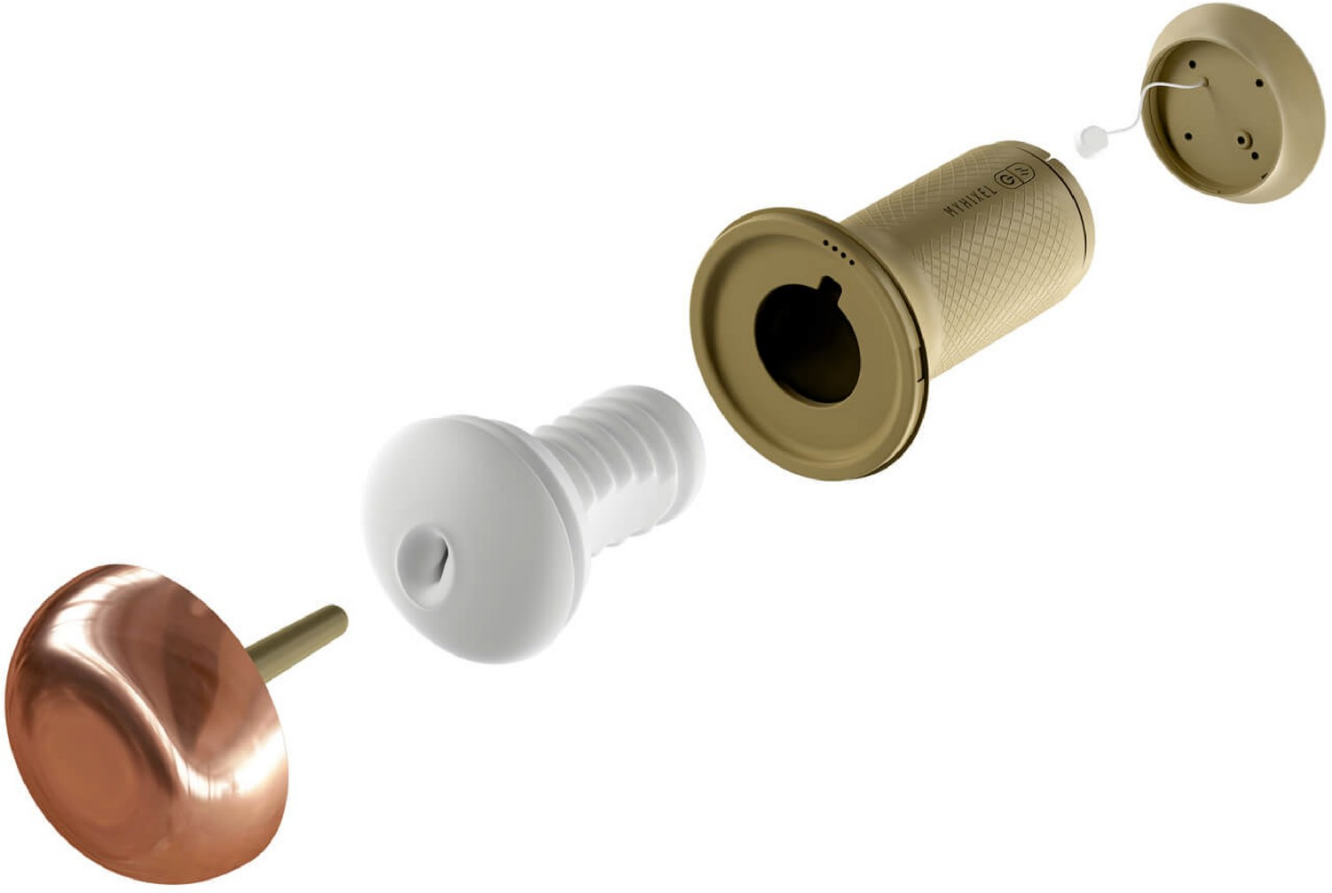
Device MYHIXEL I. International patented device SKU MX_PA_000. EAN 8437019695019. Dimension length: 113 mm. Width: 113 mm. Height: 203 mm. Diameter 91 mm. Weight 630 grams. Materials: thermoplastic elastomer, ABS silicone. Type of battery Lithium. Charger type, USB + Magnet (Waterproof).

This exercise program was developed individually with all subjects, with the only difference between both groups being the use of an electronic device to aid masturbation called Myhixel I (Fig 1), which, given its characteristics, allowed the anatomical reproduction of the vaginal introitus, reached a temperature of use between 36 and 37 degrees Celsius, and allowed the option of applying a vibration on the glans during exercises.

The SCT program consists of four different exercises and an educational session. Its objective is to provide patients with greater knowledge, awareness and control of the external sphincter of the urethra and its role in the ejaculatory reflex. The activities seek that men learn to delay the ejaculatory reflex, preventing the formation of the prostatic pressure chamber by relaxing the external sphincter during intercourse [7].

The subjects of both groups received by email an educational video presentation on the role of the external sphincter in ejaculation and the importance of its control, as well as cards and explanatory videos with each of the 4 SCT exercises along with a template for activity and IELT records. Doubts about the exercises could be consulted via email.

Once the completed records of each activity were sent, all the necessary material for the next activity was sent to them by email.

After the last record of activity, the PEDT questionnaire was administered again.

### Measurements

#### Fold increase in IELT

As the main measure, the FI in the IELT was used, which was calculated using the geometric mean (GM)* of the posttreatment IELT (period B), calculated with the recorded times of the last two weeks of treatment, divided by the geometric mean of the IELT at the beginning of the treatment that served as the baseline (period A), calculated with the times of the two weeks of records prior to the random assignment to each group, FI = (GM of 8S)/(GM of prtr) [16].

Geometric mean was calculated using mean of natural logarithm with each of the IELT calculated by the subject in each phase. The geometric mean uses its transform for its interpretation once expressed in seconds.

#### PEDT

For the diagnosis and as a secondary measure, is a tool originally validated in the Spanish-speaking population, among other. The PEDT was used, consisting of five items that evaluate difficulty delaying ejaculation, ejaculation before the patient desires, ejaculation with little stimulation, frustration related to premature ejaculation and opinion of the partner on ejaculation. The test-retest reliability is .82, and all items discriminate in a statistically significant way between patients with PE and without PE, with a cut-off score for this diagnosis [15].

### Statistical analysis

First, to verify that there was no difference between the groups, prior to treatment, the mean difference of the IELT of each group was calculated using the Mann-Whitney U test.

To analyze the difference between the two groups, a covariance analysis (ANCOVA) of the difference between GWtD and GWD of their geometric means was performed at the first 4 weeks, as well as at 8 weeks. The dependent variable is the geometric mean, the treatment was considered as a factor and the pre-IELT score as a covariate.

Afterwards, to compare how each of the groups had evolved, intragroup comparisons were made, using Wilcoxon signed rank test of related samples, between the previous values of the IELT and its geometric mean both at 4 and 8 weeks.

As a complementary measure to check the differences in the evolution of the two groups, Student’s t-test of independent samples was also calculated comparing the differences between the two groups in the FI value.

To perform the calculations, the statistical package IBM SPSS Statistics Base 22.0.

## Results

### Subjects

**Fig 2:**
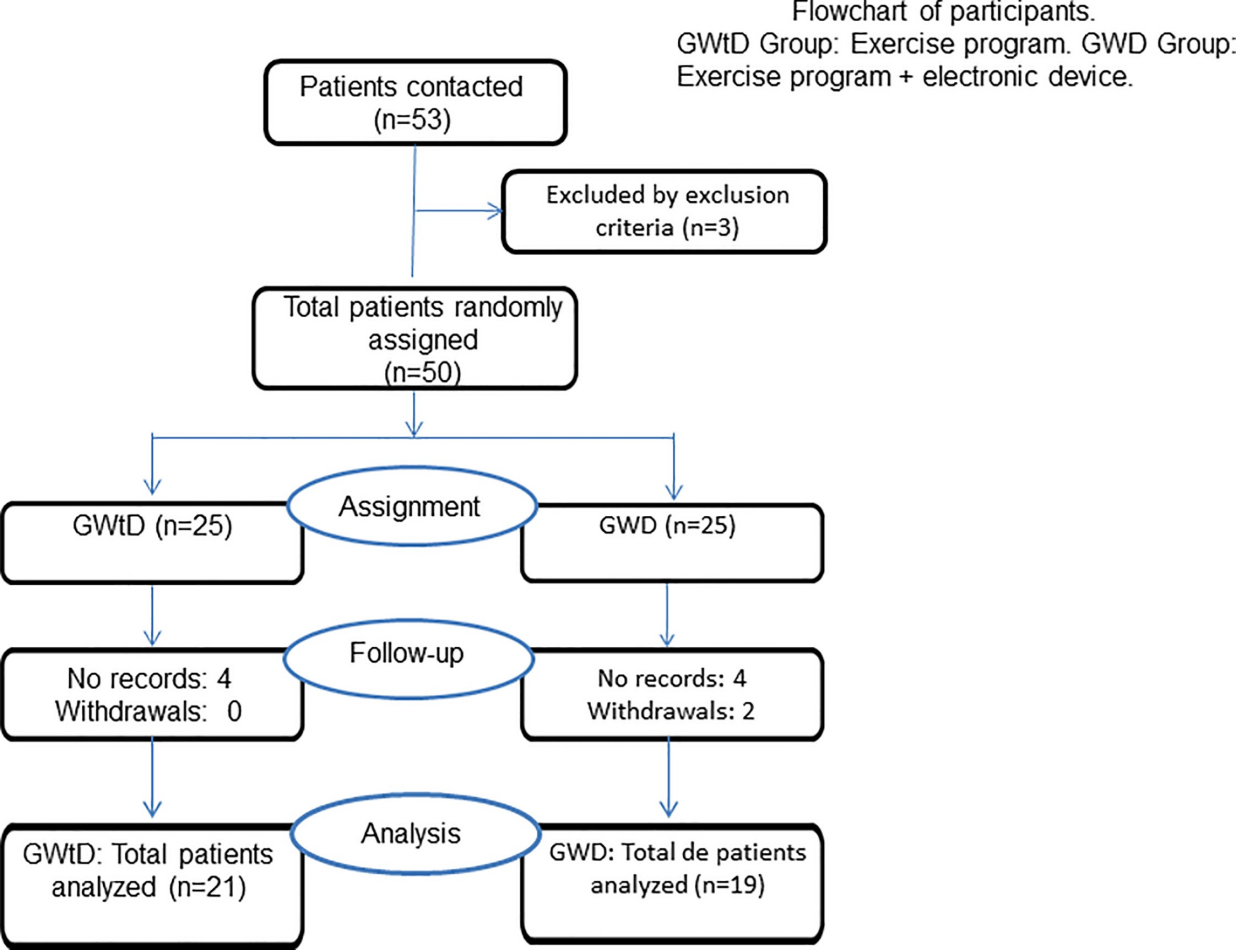
Flowchart of participants. GWtD Group: Exercise program. GWD Group: Exercise program + electronic device.

Of a total of 53 subjects who were contacted, 50 were randomly assigned by recruitment order on a 1:1 basis to the two treatment groups, 25 to the GWtD (SCT program) and 25 to the GWD (SCT program + electronic device). The experimental mortality was 24.52% since 40 subjects completed all phases of the study, and their data were analyzed. The subjects analyzed were n = 21 for the GWtD and n = 19 for the GWD (Fig 2).

### IELT and fold increase

Table 3 shows the IELT throughout the study.

**Table 3:** Intravaginal Ejaculatory Latency Times (IELT) and Fold Increase (FI) during the study.

		GWtD	GWD	*p-value*
IELT	Pretreatment(PRTR)	79.76 (33.63)	70.81 (32.89)	.093
Geometric	4 weeks(4 wk)	110.23 (54.06)	161.82 (89.67)	.111Ī .003†
mean (SD)	*p-* value (prtr/4 wk)	.001 ⱡ	.000 ⱡ	
	8 weeks (8wk)	166.28(98.37)	251.52 (110.71)	.022 Ī .001†
	*p-* value (4wk/8 wk)	.000 ⱡ	.000 ⱡ	
Fold Increase (SD)	4 weeks (prtr/4 wk)	1.43(.49)	2.71 (1.94)	.006 Ĩ
	8 weeks (prtr/8 wk)	2.09 (.72)	4.27 (2.59)	.001 Ĩ

† Covariance analysis

ⱡ Wilcoxon signed rank test related samples

Ī Mann-Whitney U test of independent samples

Ĩ Student´s t test

GWtD Group: Exercise program. GWD Group: Exercise program + device.

SD: Standard Deviation

Accepted level of significance p<0.05.

The calculation of the Mahalanobis distances indicated the existence of two outliers, one of the GWtD and another of the GWD in the variables corresponding to FI. The calculations, from now on, were carried out without these two subjects.

The Mann-Whitney U test on the IELT variable of the baseline did not show significant differences between the treatment groups prior to the intervention (p = .0.93).

Subsequently, the geometric mean for both groups of the first four weeks of treatment (4wk) was compared using the ANCOVA test, finding significant differences (F: 1.35, 10.10; p = .003) in the increase in IELT experienced by the GWD subjects compared to the GWtD subjects.

Then, the geometric mean of the first eight weeks of treatment (8wk) was calculated, and the two groups were compared using the ANCOVA test, finding significant differences (F: 1.35, 13.27; p = .001) in the increase experienced by the GWD (geometric mean = 251.52, SD = 110.71) compared to that experienced by the GWtD (geometric mean = 166.28, SD = 98.37).

The differences between the pretreatment values (PRTR) and the values at four weeks of treatment (4wk) were also analyzed for each of the groups using—Wilcoxon signed rank test. The results indicate that there are significant differences in both the GWtD (p < .05) and the GWD (p < .05) between both measures.

Next, the differences between the values corresponding to the first four weeks of treatment (4wk) and the values at the end of treatment (8wk) were also analyzed using an intragroup comparison for both.

The results showed that in both groups GWtD (p < .05) and GWD (p < .05), there were significant differences between the pretest and the posttest.

Next, the variable called FI was created, which relates the measurements obtained during the weeks prior to treatment (PRTR) with those obtained after 4 (4wk) and 8 (IELT _8wk) weeks of treatment using the expression FI = (Mean Geometric of 8wk or 4wk)/ (Geometric Mean of PRTR).

Using Student’s t-test, the FI of both groups was compared at week 4 (p = .006) and week 8 (p = .001) of treatment, finding significant differences between the GWtD and the GWD in both cases.

### PEDT

**Fig 3:**
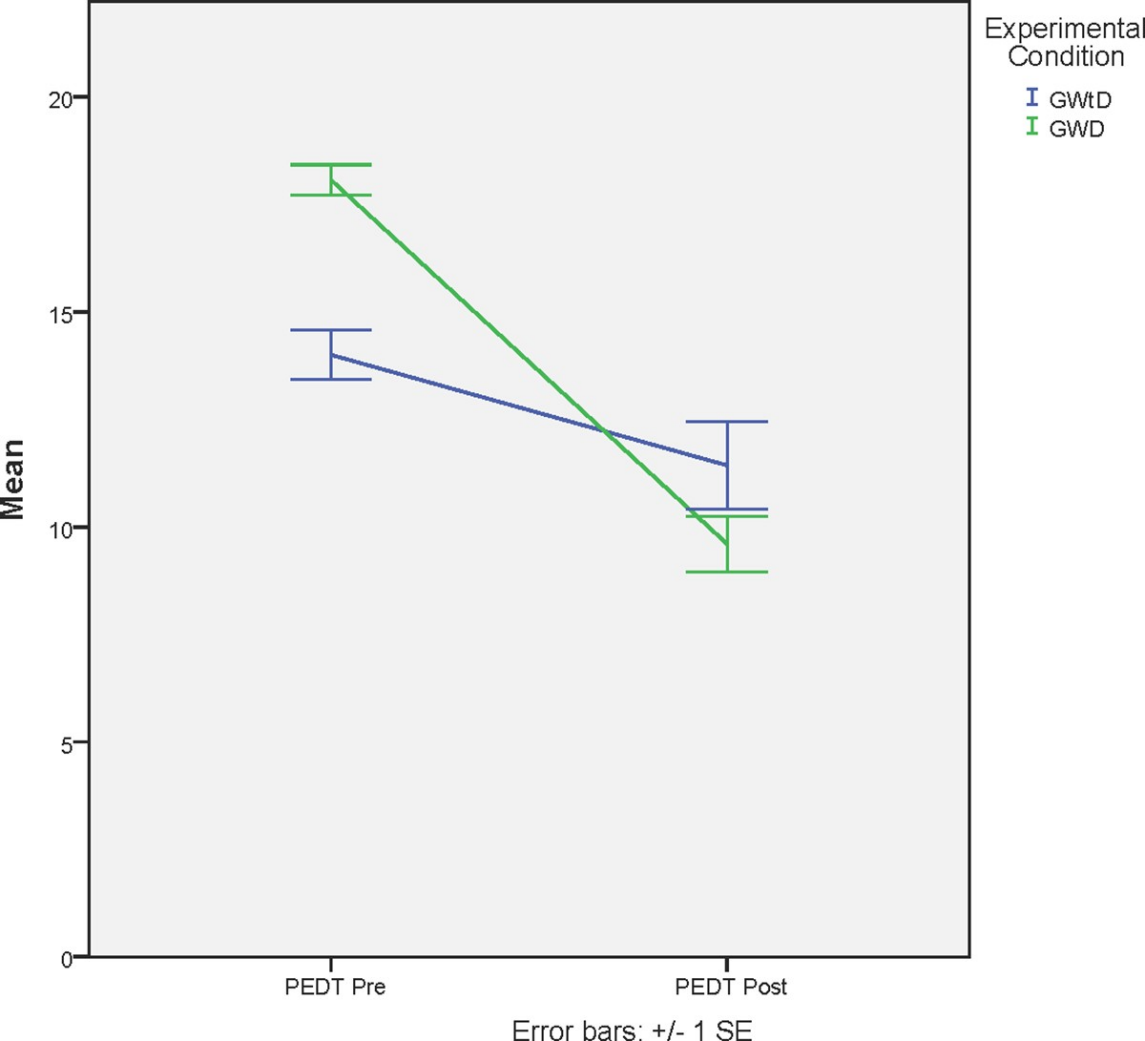
Pre-post PEDT between groups. Means in PEDT between GWD and GWtD. *Significant differences with p <0.025.

Fig 3 shows the analysis of the scores for the PEDT.

First, the T test was performed for independent samples that showed significant differences in the PEDT_PRE variable between the means of the GWtD and the GWD group (t 34 = -5.49; p < .05). However, the T test for independent samples does not show significant differences in the PEDT_POST variable between the GWD and GWtD means (t (36) = 1.48; p = .14).

Subsequently, Student’s t test was performed for related samples that showed significant differences between the pre- and post- in the GWtD (t 2 = 4.10; *p* = .001) and GWD (t 14 = 13.09; p < .05).

## Discussion

This is the second randomized clinical trial developed to measure the efficacy of SCT, a promising cognitive-behavioral treatment program. Unlike the previous clinical trial of 2019 [7] in which a device cataloged as a sex toy was used, in this case, an electronic device to aid masturbation specially designed for this program cataloged by the FDA as a class 2 medical device was used. In the previous clinical trial, Rodriguez et al. [7] also used a parallel group design with two treatment groups and measured the SCT program and the possible benefit of performing these exercises in combination with a commercial device for aiding masturbation. The SCT was designed for men to develop greater awareness and control over the external sphincter of the urethra and is based on the hypothesis that relaxation of the sphincter makes it difficult to initiate the emission phase of the ejaculatory reflex by preventing formation of the prostatic pressure chamber. The results showed significant differences between the two treatment groups at the end of the treatment in favor of the group that used the device and obtained a fold increase (FI) of 2.69. The authors attribute these differences between both groups to two factors, one to a possible effect of desensitization given the greater friction and pressure of the penis during exercises with the device and another to a greater ease of generalizing what was learned during the exercises to their sexual intercourse, since the device generated stimuli very similar during training to those produced in the penis during intercourse [7].

In that same year, Ventus [17] published another randomized clinical trial in which two parallel treatment groups and a waiting list control group were used to measure the effectiveness of vibrator-assisted start-stop exercise (VSS) programs and another version of the same program called VSS + which included psychoeducation and exercises to increase interoceptive awareness. One of the objectives was also to develop greater awareness of the pelvic floor muscles by the subjects to try to relax this as a form of control over ejaculation. In both treatment groups, they used a small hand-held vibrating device to perform stop and start exercises 3 times per week for 6 weeks, which had already been used in several case series [18]. The Swedish version of the Checklist for Early Ejaculation Symptoms (CHEES) was used as the main measure [19]. The study also includes a follow-up at 3 and 6 months. The results show significant differences in posttreatment self-reported PE scores between the two treatment and control groups, and these reductions in PE symptoms were also maintained in the follow-ups. No significant difference was found at any time during the intervention between the two treatment groups that used the VSS and VSS + programs [17]. The authors attribute these results, as in other case studies with this same device, to a desensitization effect as a mechanism of action [18].

As in the first trial that using SCT program [7], the geometric means of IELT show a superiority of the SCT treatment program in combination with the use of the device over the group that did not use the device [7].

These superiorities are at the end of the treatment at week 8, where although significant improvements are observed in the IELT for both groups over the pretest, there are still significant differences between the two.

The mean FI at the end of treatment for the GWD was clearly higher than that obtained in the previous clinical trial, in which a specific medical device was not used, ranging from 2.69 to 4.27 in the latter [7].

During the study, no side effects were observed in either of the two treatment groups, which are a great advantage in relation to first-line treatments for PE with SSRIs, since it is one of the main causes of its abandonment [20].

The differences found between both treatment groups can be attributed, as in the previous clinical trial, to the use of the device to aid masturbation. The improvement in the FI recorded in this test in relation to that of 2019 can be attributed to the characteristics of the medical device used on this occasion, specifically the interior design and the materials faithfully reproduce the vaginal introitus, reaching a use temperature of between 36 and 37 degrees Celsius, to which the inclusion of a vibratory stimulation of the penis very similar to that used by Jern in his clinical trial must be added [17].

Although the study subjects had a stable partner, the SCT program is developed individually and online without the need for collaboration on the part of the partner, which would allow it to be used in patients with PE without a stable partner or who are reluctant to include their partners in treatment or see a health professional [7].

The main limitation of this study are the use of PEDT that is a diagnostic tool and it is not designed to compare the efficacy of PE treatments other limitation is the lack of follow-up at 3 and 6 months of the treatment groups to determine the need or not to continue with the exercise to maintain the improvement found, both in the IELT and in the control perceived. Determining this issue is important since the need for continuous use is another of the main reasons for abandoning current treatments with SSRIs [20].

Although proper random assignment prevents selection bias, it does not guarantee that the groups are equivalent at baseline. Random allocation instead ensured the two groups were not systematically biased, although the score of PEDT show meaningful differences between groups (see Table 2). The online free version software GraphPad Prism used for random assignment did not allow us to conduct the Berger Exner test to determine if faulty randomization was the cause. The two groups were not systematically different (their allocation was not biased) even though they were unequal. However this may affect the internal validity and implies a further limitation of the study.

In the analysis of the data we used a per protocol approach because there were some specific reasons that led to the exclusion of ten of fifty patients from the full analysis set, as none received any treatment and no data are available after randomization.

Studies with a larger sample size and longer follow up that including new PE subtypes will be necessary to provide more evidence for this new treatment strategy for PE.

## Conclusions

We can conclude that this new cognitive-behavioral strategy for the treatment of PE, in which the SCT exercise program is combined with the use of a new electronic medical device to help masturbation, obtains satisfactory results.

## Supporting information

S1 Checklist Checklist of information to include when reporting a randomized trial. (DOC)

S1 Protocol Study protocol trial in English. ISM-SCT-2019-01.
(PDF)
