# Supplementary material for: Efficacy of Sphincter Control Training and medical device in the treatment of premature ejaculation: A multicenter randomized controlled clinical trial
Source: PLoS One. 2021 Sep 21;16(9):e0257284. doi: 10.1371/journal.pone.0257284 (PMC8454929; doi:10.1371/journal.pone.0257284)
Supplement: S1 Protocol — ISM-SCT-2019-01. (PDF) [file pone.0257284.s002.pdf]

1.PROTOCOL IDENTIFICATION

Research ID Code:
ISM-SCT-2019-01

2. PROTOCOL TITLE

SCT INVESTIGATION PROTOCOL
(Treatment for Primary Premature Ejaculation)

3. RESEARCHERS

RESEARCHERS:

Sr. Jesús Eugenio Rodríguez Martínez
Degree in Psychology. Master's Degree in Clinical Psychology.
Master’s Degree in Sexology and Human Sexuality.
Doctorate student at the Department of Health Psychology, UMH.
Director of the Sexological Institute in Murcia, Spain (ISM).

Sr. Jose Antonio Picazo Aroca.
Degree in Psychology.
Master’s Degree in Basic and Applied Neurosciences.
Ph.D. Candidate at the Health Psychology Department, UMU.
Researcher at: Instituto Sexológico Murciano (ISM).

Dr. José Antº Piqueras Rodríguez. Full Professor
Area of Personality, Evaluation and Psychological Treatment, Department of Health Psychology
University Miguel Hernández, Elche, Spain (UMH).

Dr. Juan Carlos Marzo Campos.
Full Professor.
Area of Social Psychology.
Director, Department of Health Psychology. Miguel Hernández University, Elche, Spain (UMH).

4. PROMOTER

Sr. Jesús Eugenio Rodríguez Martínez
Sexological Institute of Murcia
C/ Periodista Encarna Sanchez 22,
30007, Murcia

5. SUMMARY

5.1. *Identification of the promoter*

Sexological Institute of Murcia (ISM)
C/Periodista Encarna Sanchez, 22, P1 30007 Murcia
Teléfono: 868 94 14 18
N.I.F 48478368A

5.2. Title of Clinical Trial

A randomized closed trial (RCT) to compare the effectiveness and safety of a masturbation aid's exercise protocol as a first-line treatment for patients with primary premature ejaculation.

5.3. Protocol ID Code

Protocol ID Code: ISM-SCT-2019-01

5.4. Lead Researcher

Sr. Jesús Eugenio Rodríguez Martínez
Director, Sexological Institute of Murcia
C/ San Rafael nº2 1º L

5.5. Location of trial

The trial will be conducted in the following Clinic:

1. **Sexological Insitute of Murcia**
C/ San Rafael nº2 1º L
30007 Murcia, Spain. Tel. 868 94 14 18 RES: 40002570

5.6. Ethics committee and participating clinics.

This trial has been submitted for the approval of the Ethics Committee in the following establishment:

- Hospital José Mª Morales Meseguer – Murcia

The following clinics will take part in the trial:

- ***Sexological Institute of Murcia, Spain***

5.7. Main objective

Assess the effectiveness of a cognitive-behavioral program called Sphincter Control Training (SCT), within an app for android systems, as treatment of primary premature ejaculation in Spanish population and determine if said program is enhanced with the use of a device to help masturbation specially designed for the same..

5.8. Design

-This research project will use two experimental groups. One will be given the "SCT" therapy on its own (experimental group 1) and the other will be given the "SCT" therapy combined with the aforementioned device (experimental group 2). The subjects in both groups will experience parallel therapy with the same exercise protocol (with the only difference being that, in experimental group 2, the SCT exercises will be carried out with the device in question). The participants who meet the selection criteria will be randomly assigned an experimental group, along with its corresponding conditions.

5.9. Illness or condition under investigation

Primary Premature Ejaculation (F52.4).

5.10. *Information regarding the device under investigation and the exercise program.*

Device under investigation: Myhixel I.

Dimensions

Lenght: 4,45 Inches (113 mm)

Width: 4,45 Inches (113 mm)

Height: 8 Inches (203 mm)

Diameter 3,58 Inches (91mm)

Weight 22,22 Oz (630 grams) with lid

19,75 Oz (560 grams) while using

Materials

Thermoplastic Elestomer

ABS

Silicone

Type of Battery Lithium

Charger Type USB + Magnet (Waterproof)

DATA SHEET ANNEXED 2

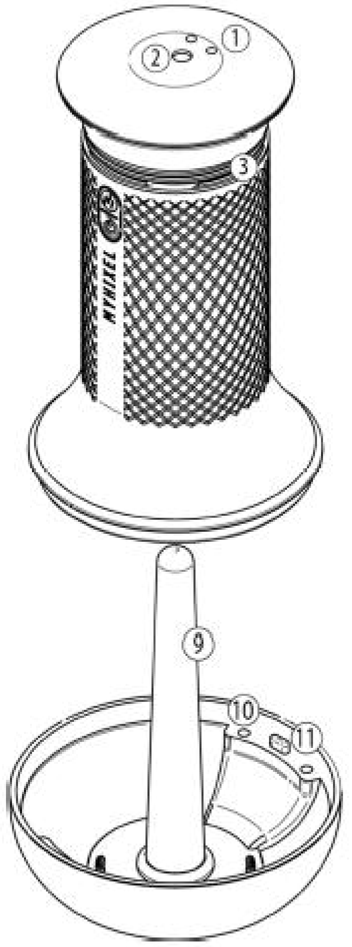

Exercise program: SCT.

The home exercise program includes 4 activities:

The first exercise called "Discovering your pelvic floor” will be carried out for one week, the second, called "Stop and Start", should be carried out for 3 weeks. The third is called "Stop and Start constant stimulation" and should be carried out for 2 weeks, and the last exercise in the program, “Stop and Start, constant stimulation with coital movements" will be carried out for a week.
Exercise information APPENDIX 3

Lubricant to be used in the trial: Myhixel lube.

Content: 50 ml (1.69 Oz)

Ingredients: Water, Propylene Glycol Hydroxycycliculose, Sodium Benzoate, Polysorbate 20, Potassium Sorbate, D-limonene, Sodium Hydroxide, Carica Papaya Fruit Extract, Chamomilla Recutita, Flower Extract, Citric Acid, Plankton Extract, Perfume, Sodium Citrate, Citral, Soluble Collagen.

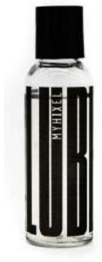

5.11. **Sample group and number of participants.**

The sample group will be heterosexual men, aged 18 or older, taken from all over the country. They all suffer from primary premature ejaculation, haven't received any treatment for the condition in the past, and have had a stable partner for at least 3 months before the start of the trial.

A sample size of 50 patients is expected. We estimate a 30% dropout or non-compliance during the study.

5.12 Schedule and proposed end date

Duration of the inclusion period: June 2019 - August 2019
Planned trial start date: July 2017
Planned trial end date: September 2017

5**.13. Financing**

Funding will be provided by the research area of the Sexological Institute, Murcia, Spain, and the NEW WELLNESS CONCEPT SL.

6. RELEVANT FINANCIAL ASPECTS OF THE TRIAL

50% of the project's funding will be provided by the research area of the Sexological Institute, Murcia, Spain, while the other 50% will be funded by the NEW WELLNESS CONCEPT SL. .

This 50 % includes the provision of 30 Myhixel I devices and 30 bottle of lubricant Myhixel lube.

MEMORIA ECONÓMICA
Código del estudio: ISM-SCT-2019-01
FINANCIAL REPORT
TRIAL CODE: ISM…

FINANCIAL REPORT ON THE PROJECTED EXPENDITURE OF THE RESEARCH PROJECT:
Title: **PROTOCOL INVESTIGATION: MYHIXEL**
Research code: **ISM-SCT-2019-01**

Lead Researcher: **Jesús Eugenio Rodríguez Martínez**
Promoter: **Sexological Institute, Murcia, Spain**

DETAILED DESCRIPTION OF PROJECTED FUNDED COSTS

**COSTS PERSONNEL:**	
1 Part-time Research Intern	400,00 €/month *
SUBTOTAL PERSONNEL:	1.200 ,00 €
**FACILITIES, MATERIALS, SOFTWARE LICENSES AND TESTS:**	
Licenses and correction kit MCMI-III, PEP, PEDT y GRISS male.<br>1 monthly license IBM SPSS statistics software.<br>MRW Courier Service ecommerce National Transport 30 kits	66,49 €<br>88,50 €<br>275,10 €
SUBTOTAL FACILITIES AND EQUIPMENT:	430,09 €
**CONSUMIBLES:**	
50 Myhixel lubricant (HOLE LOTION)<br>30 Myhixel Device	480,00 €<br>990,00€
SUBTOTAL CONSUMIBLES:	1470,00€

1

MEMORIA ECONÓMICA
Código del estudio: ISM-SCT-2019-01

**OTHER COSTS:** (specify details of costs and amounts paid)	
Domain and hosting, web landing page for trial, yocontrolo.org, Arsys S.L.U.	101,41€
Online templates Web Templates Ltd	67,00 €
SUBTOTAL OTHER COSTS:	2043,91 €
**TOTAL PROJECT BUDGET:**	**5.419,10 €**

7. CLINIC CONDUCTING THE TRIAL

The study will be conducted at the Sexological Institute, Murcia, Spain, a private health center.
Type of center and Code: C.2.2 Other health professionals.
Authorized services and code: U.900.1 OTHER UNITS PSYCHOLOGICAL ASSISTANCE Address:. Calle San Rafael, 2 Escalera 1, 1º L Murcia
Authorization of operations: 04/03/2019
Inscription in the Registry of the Health Department Nº: 40002570

8. JUSTIFICATION AND RELEVANCE OF THE TEST

Premature ejaculation (PE), understood as a lack of ejaculatory control accompanied by short (up to one minute) intravaginal ejaculatory latency (IELT) times, with minimal stimulation and before the individual desires it (Serefoglu et al., 2014). It is considered the most common male sexual dysfunction, affecting millions of men worldwide (Russo et al., 2016). Treatments for PE currently include a combination of drugs and sexual or psychological therapies, and, in particular, behavioral techniques for the man and his partner.

The psychological theories attempt to explain the etiology of PE from a point of view that includes the effect of early experience and sexual conditioning, anxiety, sexual technique, frequency of sexual activity, and psychodynamic explanations of development (e.g., Brody & Weiss, 2015). However, the biological theories have the most scientific evidence (e.g., Waldinger, 2002, Corona et al., 2008). Among these, the classic model explains the process of human ejaculation in which the emission, which forms a "pressure chamber" (created in the prostatic urethra), is followed by the expulsion (rhythmic contractions in the penile smooth muscle) of the seminal fluid (Marberger, 1974).

Although new and often better-selling drug therapies are overshadowing traditional psychological and behavioral methods in the treatment of PE, there is currently no evidence to show that there is only one physiological cause underlying PE (Althof et al., 2014). Furthermore, psychological strategies based on behavioral techniques for the treatment of PE have been shown to have had some success in alleviating this dysfunction (Althof et al., 2006).

Only a few studies have compared PE psychotherapeutic techniques, either against a control condition (currently on a waiting list) or against other psychological treatment. The lack of specific treatment protocols and research funds to conduct well-designed testing studies of these protocols has diminished the attractiveness of these approaches with respect to evolving pharmacological strategies.

This is despite the fact that several cognitive-behavioral strategies come close to meeting the criteria for empirical support.

Among them is the "start-stop" method, developed by Semans (1956) and adapted by other authors such as Glina et al (2007), which suppresses the urge to ejaculate by stopping or pausing sexual stimulation before reaching the point of imminent ejaculation by means of a “glans grip”. In addition, we know that the use of external devices can be effective in helping to control ejaculation (Rodriguez & Lopez, 2016).

This project aims to demonstrate the efficacy of Semans' adapted "start-stop" combination treatment on PE (1956) as part of an exercise program carried out with an external device similar to that used in Rodriguez & Lopez’s research (2016). The study aims to provide scientific and methodological evidence for a cognitive-behavioral treatment that offers an alternative to current pharmacological treatments for PE.

9. DESIGN AND RATIONALE

In both groups, and to enable both groups of subjects to access treatment for PE, the control will be intra-subject. The main variables and data to establish the baseline of each group will be collected during a period of a similar length to the treatment period.

-This research project will use two experimental groups. One will be given the "sct" therapy (experimental group 1) and the other will be given the "start-stop" therapy combined with the external device (experimental group 2). The two groups will receive parallel therapy with the same exercise protocol (with the only difference being that, in experimental group 2, the sct exercises will be carried out with the device in question). The participants who meet the selection criteria will be randomly assigned to an experimental group, along with its corresponding conditions.

10. MAIN OBJECTIVE

The aim of the project is to demonstrate the effectiveness on PE of the "sct" combination treatment adapted from Semans (1956) within the framework of an exercise program carried out with an new external device specific design for this method .

Its effectiveness on PE will be reflected in increased intravaginal ejaculation latency times (IELT) and subjective perception scores from baseline therapy. The increased efficacy of the combination therapy ("sct" in conjunction with external device) will be reflected in the greater increase in IELT and subjective perception scores from combination therapy over "sct" therapy. The level of scientific evidence will indicate the competitiveness and efficacy of this cognitive-behavioral psychological technique.

11. MAIN EVALUATION PARAMETER

The main evaluation parameter is the intravaginal ejaculation latency time (IELT). This is a variable that is calculated by the experimental subject himself, and is the time, in seconds, from when penetration begins until ejaculation. It is the most studied variable in research of this type (Waldinger, Zwinderman, Olivier, & Schweitzer, 2008).

Other variables taken into account are of a more psychological nature and reported by the patients themselves. On the one hand, the subjects' perception of their own ejaculation: the personal stress it causes them, their satisfaction with sexual activity/intercourse and any interpersonal difficulties relating to ejaculation. On the other hand, there are variables related to sexual satisfaction such as the patient’s perception of impotence, perception of premature ejaculation, avoidance of sexual activity/intercourse, lack of sensuality, and level of satisfaction/dissatisfaction with sexual activity/intercourse.

12. SAMPLE GROUP AND TOTAL NUMBER OF PARTICIPANTS

The subjects to be considered will be all those interested in participating who respond to the national advertising campaign that will be launched. They will be contacted by e-mail or telephone, and the questionnaires and selection records will be sent by e-mail (the criteria for inclusion in the study are defined in the following section).

All patients who come to the MSI requesting evaluation and treatment for PE and who meet the inclusion criteria will also be able to participate in the study. Subjects will be free to leave the study at any time and will not receive financial compensation for participating, although they will receive a gift at the end of the study, and a high drop-out rate is expected.

Based on randomized studies to assess the efficacy of treatments for premature ejaculation in which there was a control group, we estimate that 24 subjects per group will be needed to establish the differences between the two groups, considering a statistical power of 0.80 and alpha of $p=0.05$.

13. PATIENT INCLUSION AND EXCLUSION CRITERION

Inclusion criteria for selection will be: aged 18 or over, in a heterosexual relationship for at least the last 6 months, having a score greater than 11 on the PEDT (Premature Ejaculation Diagnostic Tool) and a mean self-reported IELT (Intravaginal Ejaculatory Latency Time) of <120 seconds.

Exclusion criteria will include: History of alcohol abuse or dependence, having received medication or psychological treatment for PE in the last 3 months, having diabetes, or a regular use of recreational drugs (except tobacco and caffeine).

14. STATISTICAL ANALYSIS

As the main evaluation parameter, the "fold change" of the IELT will be used, which will be calculated using the geometric averages of the post-treatment IELT (period B) divided by the average of the IELT at the beginning of the treatment that served as a baseline (period A).

The Mahalanobis distance test will be used to check the existence of outliers (with a Z score of > 3) and the Kolmogorov-Smirnov test to check the goodness of fit of the data and the Square Root Transformation in those cases where the assumption of normality is not met.

The Greenhouse-Geisser method will be used to correct for degrees of freedom when the sphericity assumption is not met.

The MANOVA contrast will be performed for the demographic variables, and, presumably, we will find significant differences in variables such as age.

For the contrasts, ANCOVAS of independent measurements will be used, with the variables that have come out as significant in the MANOVA contrast as covariates, to check the differences between the two experimental groups and the equality between baselines in each of the study variables. And, similarly, ANCOVAS of repeated measurements to check the existence of the benefits of each therapy on the baseline.

All p values should be bilateral and the significance level used < 0.05. The IBM SPSS Statistics version 22 statistical package should be used.

15. ETHICAL CONSIDERATIONS

This study should be conducted in accordance with the protocol and Good Clinical Practice (GCP) guidelines, as described in the ICH Harmonised Tripartite Guidelines for Good Clinical Practice 1996.

By signing this protocol, the investigator agrees to follow the instructions and procedures described in the protocol and will therefore comply with the principles of Good Clinical Practice on which it is based, bearing in mind the Declaration of Helsinki http://www.unav.es/cdb/ammhelsinki2.html.

All patients will go through a therapeutic condition. In the case of "sct" therapy, the efficacy is proven by previous research and trials (Glina et al., 2007. Rodriguez et al., 2019) and, in the case of combined therapy, the health risk is minimal and the probability of its efficacy is at least equal to that of "start-stop" therapy since, in this case, the former encompasses the latter.
The data collected will be subject to the data protection law: Organic Law 15/1999 of 13 December on the Protection of Personal Data (LOPD). And the informed consent is collected in accordance with the Royal Decree 223/2004 of February 6.

16. DURATION OF THE TREATMENT

The duration of the treatment will be 7 weeks, during which the previously-explained exercises will be carried out.

17. EVALUATION AND SAFETY OF THE TRIAL

The products to be used in the research will be safe and their responsible use will not produce any adverse reactions.

A personal data file will be created, to be owned by the Sexological Institute of Murcia; its sole purpose will be the collection of the data from the trial and its subsequent study.
A backup copy of the data and its recovery procedures will be stored separately to the computer system involved in the trial.

Each subject will be assigned a random, statistically-generated, numerical or alphanumerical code.

The person responsible for the file and/or its processing will guarantee that only he or she has access to the data and its subsequent processing.

Confidential data will be encrypted with an encryption algorithm and a key which will ensure that the data is unreadable without prior knowledge of the key.

Non-automated files shall be stored in cabinets or document filing cabinets, which, in turn, shall be stored in an area with access protected by lockable doors.

The lubricants used are made from harmless ingredients. There should be no problems in case of ingestion. However, in case of irritation the patients will be instructed to stop using it and will be excluded from the investigation. Participants will be informed about the ingredients before use and will all be instructed to carry out a small tolerance test on their arm before use in the genital area.

18. SCHEDULE AND PROPOSED END DATE

The selection and inclusion period for patients to participate in the study is set between June 2019 and August 2019.

The planned date for the commencement of the trial and the treatment is the end of June and July 2019. The planned date for the completion of the exercise program in both groups is predicted to be at the end of August 2019.

19. INTERVIEWS, QUESTIONNAIRES OR INSTRUMENTS TO BE USED IN THE TRIAL

The IELT measurements will be collected by self-registration after each week of treatment (APPENDIX I).

In order to collect the information regarding the perception of ejaculation, the Premature Ejaculation Profile (PEP) will be used (Patrick et al., 2009), a self-reported instrument consisting of four measurements (perceived control over ejaculation, personal stress related to ejaculation, satisfaction with sexual relationships, and interpersonal difficulty related to ejaculation). These will give an overall profile and score (Patrick et al., 2009), where the lowest scores express the lowest performance. The reliability coefficient between ranges from 0.66 to 0.83 and its validity was contrasted with dapoxetine and Intravaginal Ejaculation Latency Times (IELT). This instrument was validated in American and European trials, with people from Germany, France, England, Italy and Poland involved (Giuliano et al., 2008) although it has been used also in the Spanish community (Bar-Or, Salottolo, Orlando, Winkler, & Group, 2012; Buvat, Tesfaye, Rothman, Rivas, & Giuliano, 2009).

The Premature Ejaculation Diagnostic Tool (PEDT) will be used for the diagnosis. It takes five measurements in order to evaluate difficulty in delaying the ejaculation, ejaculation occurring before the patient wanted it, ejaculation with little stimulation, frustration related to premature ejaculation, and the couple's opinion about ejaculation. The test-retest reliability is 0.82 and all values statistically distinguish and differentiate statistically between PE and non-PE patients.

The Golombok-Rust Inventory of Sexual Satisfaction (GRISS) will also be applied. This is a brief 28-question questionnaire to assess the existence and severity of sexual problems in both members of a heterosexual relationship (Rust & Golombok, 1985, 1986). This questionnaire provides separate scores for men and women on 3 shared scales (avoidance, non-sensitivity, and dissatisfaction) and 6 gender-specific scales (impotence, premature ejaculation, anorgasmia, vaginismus, infrequency, and lack of communication) with acceptable values of reliability and validity (Rust & Golombok, 1985, 1986). The instrument was validated in the English community and is already being used in Spain in the official Master's degree in Sexology at the University of Almeria (García González, 2013; López Lorente, 2013).

The external masturbation aid will be the Myhixel I device manufactured by the company , NEW WELLNESS CONCEPT SL. (ANNEXED 2).
The Lubricant to be used in the trial will be Myhixel lube.

20. BIBLIOGRAPHICAL REFERENCES

Althof, S. E., McMahon, C. G., Waldinger, M. D., Serefoglu, E. C., Shindel, A. W., Adaikan, P. G., Torres, L. O. (2014). An Update of the International Society of Sexual Medicine's Guidelines for the Diagnosis and Treatment of Premature Ejaculation (PE). Sexual Medicine, 2(2), 60–90. https://doi.org/http://dx.doi.org/10.1002/sm2.28

Althof, S., Rosen, R., Symonds, T., Mundayat, R., May, K., & Abraham, L. (2006). Development and Validation of a New Questionnaire to Assess Sexual Satisfaction, Control, and Distress Associated with Premature Ejaculation. The Journal of Sexual Medicine, 3(3), 465–475.

Bar-Or, D., Salottolo, K. M., Orlando, A., Winkler, J. V, & Group, T. O. D. T. S. (2012). A Randomized Double-blind, Placebo-controlled Multicenter Study to Evaluate the Efficacy and Safety of Two Doses of the Tramadol Orally Disintegrating Tablet for the Treatment of Premature Ejaculation Within Less than 2 Minutes. European Urology, 61(4), 736–743.

Brody, S., & Weiss, P. (2015). Erectile Dysfunction and Premature Ejaculation: Interrelationships and Psychosexual Factors. The Journal of Sexual Medicine, 12(2), 398–404. https://doi.org/http://dx.doi.org/10.1111/jsm.12738

Buvat, J., Tesfaye, F., Rothman, M., Rivas, D. A., & Giuliano, F. (2009). Dapoxetine for the Treatment of Premature Ejaculation: Results from a Randomized, Double-Blind, Placebo-Controlled Phase 3 Trial in 22 Countries. European Urology, 55(4), 957–968. https://doi.org/10.1016/j.eururo.2009.01.025

Corona, G., Jannini, E. A., Mannucci, E., Fisher, A. D., Lotti, F., Petrone, L., … Forti, G. (2008). Different Testosterone Levels are Associated with Ejaculatory Dysfunction. The Journal of Sexual Medicine, 5(8), 1991–1998.

García González, S. (2013). Desire, Autostimulation, Satisfaction and Sexual Fantasies in People with Special Needs. University of Almería. Retrieved from http://repositorio.ual.es/bitstream/handle/10835/2542/Trabajo.pdf?sequence=6
Giuliano, F., Patrick, D. L., Porst, H., La Pera, G., Kokoszka, A., Merchant, S., … Polverejan, E. (2008). Premature Ejaculation: Results from a Five-Country European Observational Study. European Urology, 53(5), 1048–1057. https://doi.org/10.1016/j.eururo.2007.10.015

Glina, S., Abdo, C. H., Waldinger, M. D., Althof, S. E., Mc Mahon, C., Salonia, A., & Donatucci, C. (2007). Premature Ejaculation: A New Approach by James H. Semans. Journal of Sexual Medicine, 4(4), 831.

López Lorente, A. M. (2013). Dependent Relationships and their Influence on the Sexual Satisfaction of Ill Alcoholics, University of Almería. Retrieved from: http://repositorio.ual.es/bitstream/handle/10835/2365/Trabajo.pdf?sequence=1&isAllowed=y

Marberger, H. (1974). The Mechanisms of Ejaculation. In Physiology and Genetics of Reproduction (pp. 99–110). Springer.
Patrick, D. L., Giuliano, F., Ho, K. F., Gagnon, D. D., McNulty, P., & Rothman, M. (2009). The Premature Ejaculation Profile: Validation of Self-Reported Outcome

Measures for Research and Practice. BJU International, 103(3), 358–364. https://doi.org/10.1111/j.1464-410X.2008.08041.x

Rodríguez, J. E., & López, A. (2016). Male Masturbation Device for the Treatment of Premature Ejaculation. Asian Pacific Journal of Reproduction, 5(1), 80–83.
Russo, A., Capogrosso, P., Ventimiglia, E., La Croce, G., Boeri, L., Montorsi, F., & Salonia, A. (2016). Efficacy and Safety of Dapoxetine in Treatment of Premature Ejaculation: An Evidence-ba sed Review. International Journal of Clinical Practice, 70(9), 723–733.

Rodriguez JE, Marzo JC, Piqueras JA. Efficacy of Sphincter Control Training (SCT) in the treatment of premature ejaculation, a new cognitive behavioral approach: A parallel-group randomized, controlled trial. PLoS One 2019 Feb 26;14(2):e0212274.

Rust, J., & Golombok, S. (1985). The Golombok-Rust Inventory of Sexual Satisfaction (GRISS). British Journal of Clinical Psychology, 24(1), 63–64. https://doi.org/10.1111/j.2044-8260.1985.tb01314.x

Rust, J., & Golombok, S. (1986). The GRISS: A Psychometric Instrument for the Assessment of Sexual Dysfunction. Archives of Sexual Behavior, 15(2), 157–165.
Semans, J. H. (1956). Premature Ejaculation: A New Approach. Southern Medical Journal, 49(4), 353–358.

Serefoglu, E. C., McMahon, C. G., Waldinger, M. D., Althof, S. E., Shindel, A., Adaikan, G., … Torres, L. O. (2014). An Evidence-Based Unified Definition of Lifelong and Acquired Premature Ejaculation: Report of the Second International Society for Sexual Medicine Ad Hoc Committee for the Definition of Premature Ejaculation. Sexual Medicine, 2(2), 41–59. https://doi.org/http://dx.doi.org/10.1002/sm2.27

Waldinger, M. D. (2002). The Neurobiological Approach to Premature Ejaculation. The Journal of Urology, 168(6), 2359–2367.

Waldinger, M. D., Zwinderman, A. H., Olivier, B., & Schweitzer, D. H. (2008). Geometric Mean IELT and Premature Ejaculation: Appropriate Statistics to Avoid Overestimation of Treatment Efficacy. The Journal of Sexual Medicine, 5(2), 492–499

Collaborator’s signature:
Instituto Sexológico Murciano

INSTITUTO SEXOLOGICO MURCIANO
N.R.S 20300055
Tel [illegible] 94 14 1[illegible]
[illegible]

Lead Researcher’s signature
D. Jesús Eugenio Rodríguez Martínez

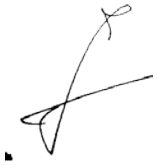

Annexed 1

Data collection forms

REGISTER OF EJACULATION TIMES

Instituto Sexológico Murciano

WEEK:

NAME:

	MONDAY	TUESDAY	WEDNESDAY	THURSDAY	FRIDAY	SATURDAY	SUNDAY
**MASTURBATION** — Record **the time you take to ejaculate** when **masturbating**							
**PENETRATION INTERCOURSE** — Record **the time you take to ejaculate** after **penetration**							

Annexed 2

ANEXO 2

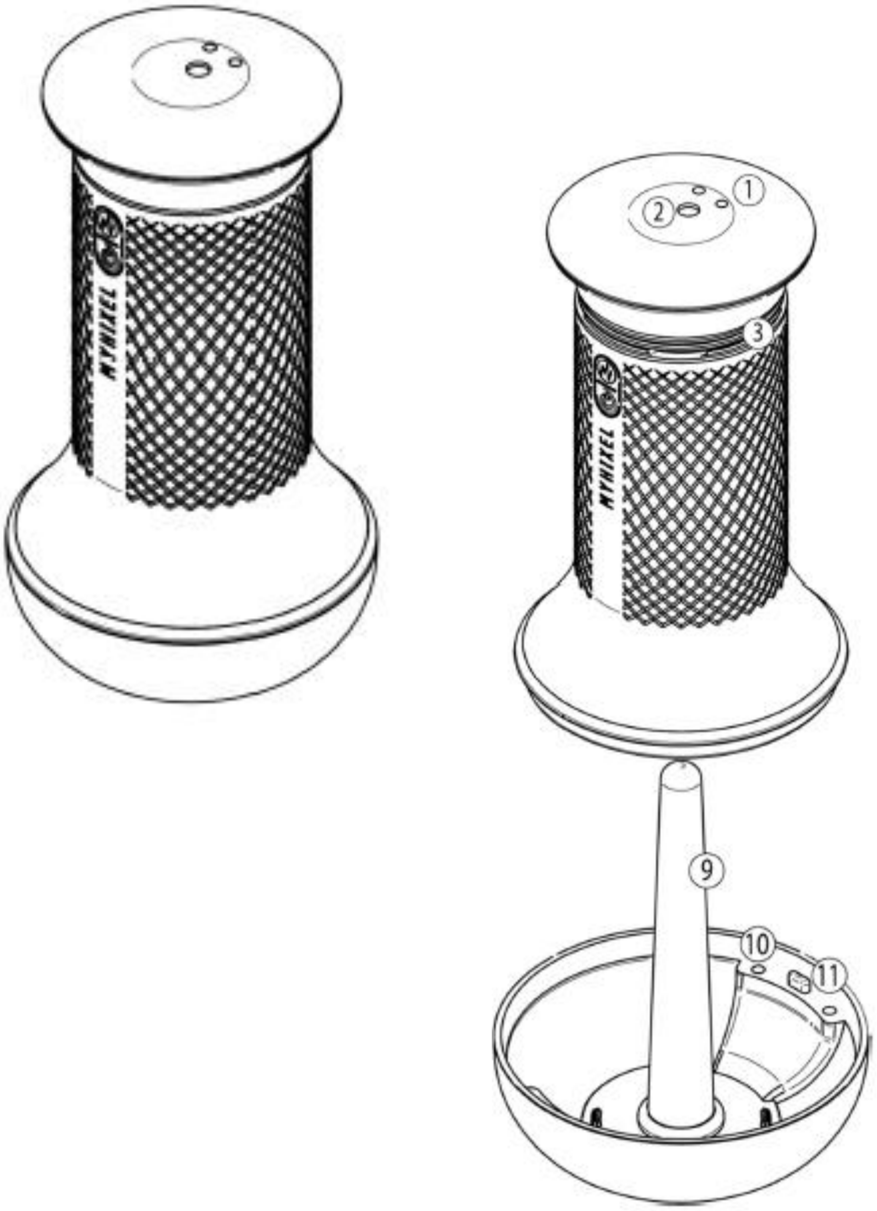

PARTES.

1 – Pines magnéticos de carga

2 – Rosca hembra para aplique de manos libres.

3 – Rosca reguladora de succión.

4 - Botonera para control de vibración y calefacción.

5 – Conexión con resistencia calefactora

6 – Orificio de entrada de la manga

7 – Motor vibrador

8 – Bolsillo para albergar el motor vibrador.

9 – Palo calefactor.

10 y 11 – Sistema de imanes para asegurar el cierre.

[redacted]

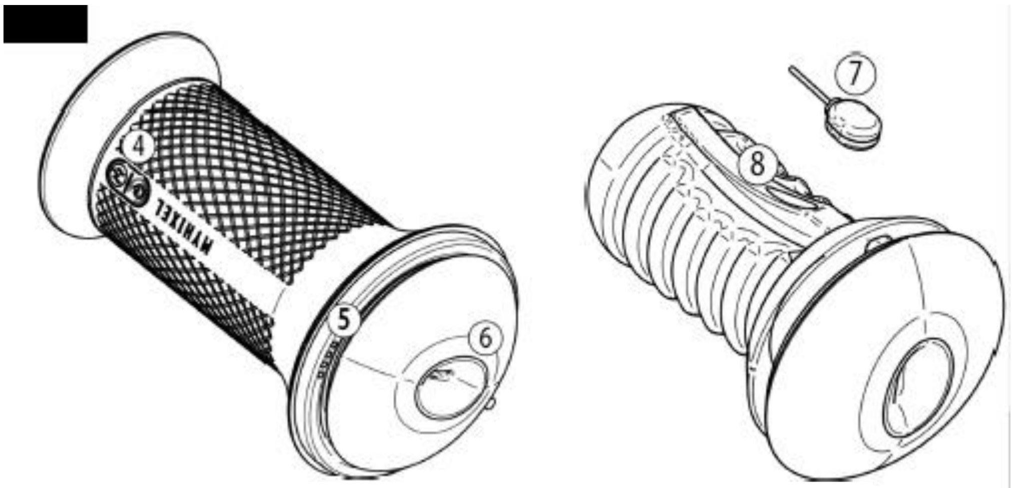

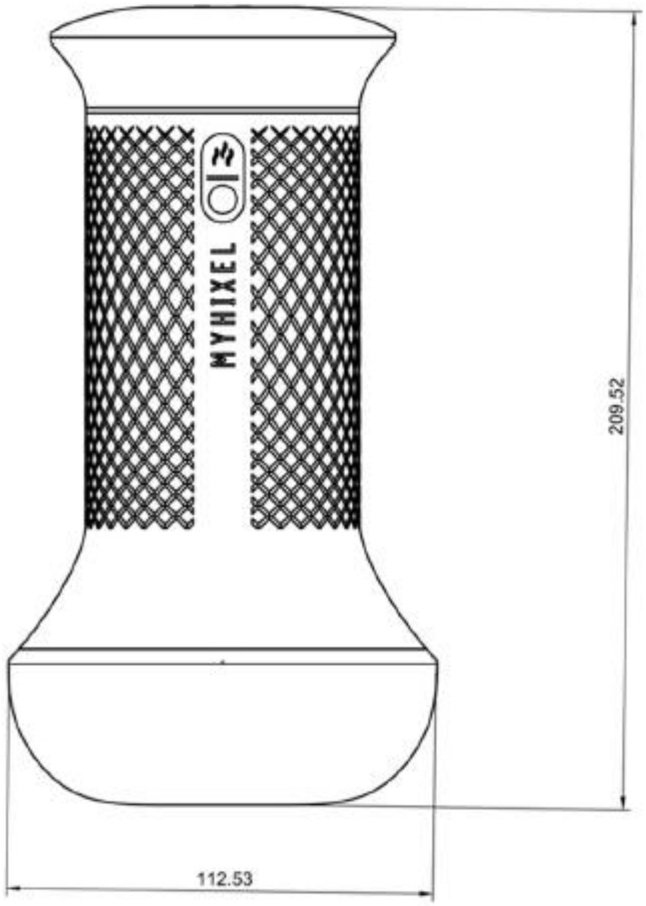

DIMENSIONES PRINCIPALES

MATERIALES Y PESO

PARTE	MATERIAL
MANGA	TPE (Elastómero termoplástico)
CUERPO	ABS
PALO CALEFACTOR	
RECUBRIMIENTO DEL CUERPO “SOFT TOUGHT”	PDMS (“Silicona soft touch”)

PARTE	PESO (gramos)
MANGA	340
MYHIXEL I (con manga)	670

MYHIXEL I (en uso, sin calefactor)	560

ELECTRONICA Y FUNCIONES

PARTE	MATERIAL
BATERÍA	POLÍMERO DE LITIO (650 Ma 3.7 V)
MOTOR VIBRADOR	~2 V (3.5 mm y 100 HZ)
CALEFACCIÓN	RESISTENCIA EN POLIAMIDA
CARGA	PINS MAGNÉTICOS + USB

**1**	**2**	**3**	**4**	**5**	**6**
LED 1+2	LED 1+2	LED 1+2	LED 1	LED 1	LED 2
**CARGANDO**	**CARGADO**	**BATERÍA BAJA**	**CALENTANDO**	**TEMPERATURA ALCANZADA**	**VIBRACIÓN**
**Leds ON fijo**	**Leds OFF**	**Los leds parpadean alternativamente**	**Led ON**<br>**Latente**<br>**(ver link adjunto)**	**Led OFF**	**Led On**<br>**Durante 10 sg y luego OFF**

COLOR LEDS (1 y 2): **BLANCO CÁLIDO**

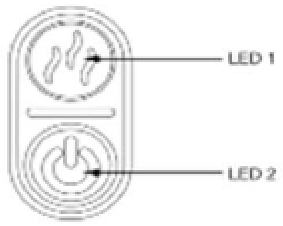

APPENDIX 3

SCT Exercise Program
PATIENT FORMS

In the first week, we're going to start with what we call the **Stop and Go Flip Hole Program**.

OBJECTIVES

1. To become aware of the role of the pelvic floor in the orgasmic response.
2. To identify the pattern of muscle tension in the genital area that's necessary to ejaculate.
3. To identify the voluntary nature of that pattern of muscular tension that's necessary to ejaculate.

KEY POINTS

Perform the activity only after you've watched the video that corresponds to the activity Discovering the Pelvic Floor.

This activity is carried out individually.

Before carrying out the activity, the participant must understand its theoretical content.

It should be carried out a minimum of 4 times or until the participant is able to reach the objectives of this first week.

This activity isn't just about masturbation, but rather about becoming aware of the role of the pelvic floor.

STEPS TO FOLLOW

Find a quiet moment where you can't be interrupted. Turn off your phone, TV, and radio, and don't let children or other people living with you distract you.

Before you start, apply a few drops of lubricant to the Flip Zero device, which you will find in the kit. Start to caress your penis, slowly and progressively, until you get an erection. From this point you will start to masturbate using the Flip Zero device, paying attention to the changes that occur around the genital area and in the nearby muscles.

It's important that you try to identify how muscle tension increases in the genital area and muscles near it as your excitement increases. You must consciously identify these points of muscular tension or tightness in the legs, abdomen, buttocks and especially in the pelvic floor (under the testicles and in the anal sphincter).

Continue masturbating until you ejaculate and observe that in order to provoke the ejaculatory reflex, you don't only need to stimulate the penis, but you also need to create muscular tension in the area, and especially in the pelvic floor.

We can see, therefore, that muscular tension in the pelvic floor area is a necessary condition for ejaculation, and it is also a voluntary factor. This muscular group is the key to either delaying or accelerating the ejaculatory reflex.
If this muscle group, known as the pelvic floor, and especially the anal sphincter and the urethra, are not in tension, the ejaculation reflex cannot occur.

During the following three weeks we will continue with what is called the **Start-Stop Program.**

OBJECTIVES

1. To get to know the physiological changes in a complete male sexual response cycle.
2. To identify the changes in the musculature of the genital area in the pre-orgasmic phase.
3. To identify the voluntary nature of the muscular tension that initiates the pre-orgasmic phase.
4. To begin to exert control over this musculature involved in the pre-orgasmic phase.

KEY POINTS

Only perform this activity after having identified (in activity 1) the muscle tension in the genital area prior to ejaculation and having understood the role that the pelvic floor plays in the orgasmic response.

The activity is performed individually, trying to ensure that it lasts at least 5 minutes.

The participant must understand the theoretical content of the activity before performing it.

It will be performed as many times as necessary until the man is able to achieve the objectives of this second activity; we recommend a minimum of 4 times per week.

Again, this is not simply about masturbation, it's a self-control training activity.

STEPS TO FOLLOW

Find a quiet time and place where you won't be interrupted. Turn off your phone, TV or radio, and don't let children or other people living with you come near the bedroom.

Start to caress your penis, slowly and progressively, until you get an erection. You will then start masturbating with the lubricated Flip Zero device, paying attention to the changes in your genital area.

It's important that you try to identify how, as the excitement increases, the muscle tension in the genital area and muscles close to it increases. You must consciously identify these points of muscle tension or tightness in the legs, abdomen, buttocks and, especially, the pelvic floor.

Continue masturbating until you feel that you're about a minute or two away from ejaculation. At this point, stop stimulating yourself and try to identify how you have triggered the ejaculatory reflex, by generating muscle tension in the pelvic floor area and around the genitals.

Then, within a minute of stopping the stimulation, practice releasing the tension in that area, relaxing it, and paying special attention to the anal sphincter and urethra, which should become tension-free.

When you start stimulating yourself again, you should notice that, if you've really managed to release the tension, then you'll be moving away from a point of climax and that you're no longer so close to ejaculating. This process of stopping the stimulation and starting again should be done at least four times in each activity. Once you've done this at least four times, you will then allow yourself to proceed towards ejaculation, allowing the full muscle tension to occur in the genital area.

In the fifth week we're going to continue the process with what we call the **Stop and Go Program without interrupting stimulation.**

OBJECTIVES

1. To get to know the physiological changes of the complete male sexual response cycle.
2. To identify the physiological changes of the pre-orgasmic phase.
3. To identify the voluntary nature of the pre-orgasmic phase.
4. To strengthen the control of the pre-orgasmic phase using the pelvic floor muscles in conditions similar to intercourse.

KEY POINTS

The activity should only be carried out after the participant understands the role the pelvic floor plays in the orgasmic response, as well as having achieved the objectives of the previous weeks.

The activity is to be carried out individually.

Before carrying out the activity, the man must understand its theoretical content and watch the accompanying video.

It should be performed as many times as necessary until the man is able to reach the objectives of this third activity. We recommend a maximum of 5 times a week.

It isn't simply a question of masturbating, but, rather, of a guided self-control training activity.

STEPS TO FOLLOW

Find a quiet time and place where you won't be interrupted. Turn off your phone, TV or radio, and don't let children or other people living with you come near the bedroom.

Start to caress your penis, slowly and progressively, until you get an erection. You will then start masturbating with the well-lubricated Flip Zero device, paying attention to the changes in your genital area and surrounding areas.

It's important that you try to identify how, as the excitement increases, the muscle tension in the genital area and muscles close to it increases. You must consciously identify these points of muscle tension or tightness in the legs, abdomen, buttocks and, especially, the pelvic floor.

Continue masturbating until you feel you're a minute or two away from ejaculation if you continue as you are. At that point, while continuing to stimulate your penis with the masturbation device, and without changing the rhythm, focus your attention on the muscle tension in the pelvic floor area and around the genitals.

Then, and without any interruption in the stimulation, practice relaxing the area and releasing the tension, giving special attention to the anal sphincter and the urethra, which should be tension-free. To test whether this is the case, you can try to simulate urination, and this will relax the sphincters automatically.

If you have managed to release the tension, then you will notice then you'll be moving away from a point of climax and that you're no longer one or two minutes away from ejaculating. However, you need to ensure that you're constantly stimulating your genital area with the device.

This process of delaying the ejaculatory reflex should be performed at least four times in each masturbation activity. Once you’ve done this, you will finish the process letting the muscle tension process continue and ejaculating into the device.

In the seventh week, we'll continue the process with what we call the **Start-Stop Zero Program with small interruptions and the simulation of penetration through hip movements.**

OBJECTIVES

1. To get to know the physiological changes during the complete male sexual response cycle.
2. To identify the body's hip movements without the need to tense the pelvic floor area.
3. To identify the voluntary character of the aforementioned muscular tension during the pre-orgasmic phase.
4. To strengthen the control of the pre-orgasmic phase using the pelvic floor muscles in a similar way to intercourse and with hip movements.

KEY POINTS

The activity should only be performed once the participant has understood the role of the pelvic floor in the orgasmic response, as well as having successfully achieved the objectives of the previous weeks.

The activity should be performed individually and without the use of the hands to masturbate.

The participant should learn to move his hips without tensing the pelvic floor muscles.

The process should be carried out as many times as necessary until the man is able to reach the objectives of this seventh week; we recommend carrying out the process a maximum of 5 times in the week.

Again, this isn't simply a question of masturbating or reaching climax, but a guided training activity in muscle self-control.

STEPS TO FOLLOW

Find a quiet moment and place where you won't be interrupted. Use audiovisual material of a sexual nature to focus your attention and cause a higher level of excitement, as well as making you less sensitive to internal sensations.

You should then begin to caress the penis, slowly and progressively, until you get an erection. At that point, you should start to use the Flip Zero device, sufficiently lubricated, being aware of everything that's happening in your body at the same time. You should do this in a standing or kneeling position, using thrusting forward hip movements with the device in position, simulating penetration, and not using your hands.

Continue to perform these movements until you feel that you're a minute or two away from ejaculation, at which point, and without removing your penis from the masturbation device, focus on your muscle tension in the pelvic floor area and around the anal sphincter, with special attention to the buttocks. Next, practice releasing the tension in that area to leave it completely relaxed, giving special attention to the anal sphincter, buttocks, and pelvic floor muscles, which should be free of tension. In order to do this, you can try to simulate urination to relax these sphincters voluntarily.

If you've been able to release the tension successfully, then you'll notice that you're moving away from the point of climax and that you're no longer one or two minutes away from ejaculation. Remember that you must do all of this without removing your penis from the lubricated device. The key to controlling this tension is to move your hips as if you were hula-hooping, and, in this way, you can develop speed without causing muscle tension.

This process of delaying the ejaculatory reflex should be carried out as many times as necessary in each masturbation activity. The total duration of the exercise should be no less than 15 minutes, and, after this time, you can then allow the muscle tension to continue in the buttocks and anal sphincter and ejaculate into the device.
